# Decreased hippocampal brain‐derived neurotrophic factor and impaired cognitive function by hypoglossal nerve transection in rats

**DOI:** 10.1111/jcmm.13284

**Published:** 2017-08-02

**Authors:** Doyun Kim, Sena Chung, Seung‐Hyun Lee, Se‐Young Choi, Soung‐Min Kim, JaeHyung Koo, Jong‐Ho Lee, Jeong Won Jahng

**Affiliations:** ^1^ Department of Oral and Maxillofacial Surgery Dental Research Institute Seoul National University School of Dentistry Seoul Korea; ^2^ Department of Brain Science Daegu Gyeongbuk Institute of Science & Technology Dae Gu Korea; ^3^ Department of Physiology Dental Research Institute Seoul National University School of Dentistry Seoul Korea

**Keywords:** hippocampus, learning and memory, tongue

## Abstract

The hypoglossal nerve controls tongue movements, and damages of it result in difficulty in mastication and food intake. Mastication has been reported to maintain hippocampus‐dependent cognitive function. This study was conducted to examine the effect of tongue motor loss on the hippocampus‐dependent cognitive function and its underlying mechanism. Male Sprague Dawley rats were subjected to the initial training of Morris water maze task before or after the bilateral transection of hypoglossal nerves (Hx). When the initial training was given before the surgery, the target quadrant dwelling time during the probe test performed at a week after the surgery was significantly reduced in Hx rats relative to sham‐operated controls. When the initial training was given after the surgery, Hx affected the initial and reversal trainings and probe tests. Brain‐derived neurotrophic factor (BDNF) expression, cell numbers and long‐term potentiation (LTP) were examined in the hippocampus on the 10^th^ day, and BrdU and doublecortin staining on the 14^th^ day, after the surgery. Hx decreased the hippocampal BDNF and cells in the CA1/CA3 regions and impaired LTP. BrdU and doublecortin staining was decreased in the dentate gyrus of Hx rats. Results suggest that tongue motor loss impairs hippocampus‐dependent cognitive function, and decreased BDNF expression in the hippocampus may be implicated in its underlying molecular mechanism in relation with decreased neurogenesis/proliferation and impaired LTP.

## Introduction

Neuronal circuits in taste sensory system are closely connected with other nerve systems for efficient handling of taste information; that is, taste sensory information that reached the nucleus tractus of solitarius is principally relayed to the gustatory cortex *via* the parabrachial nucleus, but also targets other areas of the brain, such as the cerebral cortex, hippocampus, amygdala, hypothalamus and nucleus accumbens for better storage or recall of taste memory or the innate and instinctive response such as preference and aversion 1, 2, 3. Thus, it is expected that the deprivation or disruption of taste sensory relays may affect the function of those brain regions. For example, it has been reported that decreased responses in the reward network including the nucleus accumbens to palatable food may be a trait marker of vulnerability to depression 4, 5.

Lingual sensory nerves can be damaged by dental surgery or trauma such as physical irritation, radiation, chemotherapy or viral infection. Some patients in dental clinics appeal not only altered taste perception but also negative emotion after lingual nerve damages. Indeed, oral sensory deficits in rodents with lingual nerve damages increased anxiety‐ and depression‐like behaviours 6 and affected food intake 7. It has been demonstrated that mastication is of great importance for oral sensory input to the hippocampus for preserving and promoting the cognitive function. Accumulating evidence indicates that mastication maintains hippocampus‐dependent cognitive function, and impaired masticatory function, that is, impaired oral motor stimulation, causes morphological and functional alterations of the hippocampus inducing impairments in hippocampus‐dependent learning and memory 8. Tongue movements are essential for an effective mastication and deglutition. The hypoglossal nerve controls tongue movements and damages of it result in difficulty mastication and food intake. Thus, it has been suggested that impaired tongue movements may affect hippocampus‐dependent cognitive function. In this study, we have examined the hippocampus‐dependent cognitive function in rats with bilateral transection of hypoglossal nerves (Hx). Behavioural assessment was performed with Morris water maze task, and its underlying mechanism was investigated with examining the hippocampal physiology, neurogenesis and related gene expressions.

## Materials and methods

### Animals

Male Sprague Dawley rats (8 weeks of age) were purchased (Orient bio Co, Seongnam, Korea) and cared in the animal facility at Seoul National University. Handling and care of the animals were in accordance with the Guideline for Animal Experiments, 2000, edited by the Korean Academy of Medical Sciences, which is consistent with the National Institutes of Health (NIH) Guidelines for the Care and Use of Laboratory Animals, revised 1996. All animal experiments were approved by the Committee for the Care and Use of Laboratory Animals at Seoul National University.

### Surgery

Rats were anesthetized with an intraperitoneal injection of a 4:1 mixture of ketamine hydrochloride (100 mg/kg, Ketara^®^, Yuhan, Korea) and xylazine hydrochloride (25 mg/kg, Rumpun^®^, Bayer, Korea). A ventral‐medial incision was made in the neck after cleaning the surgical field, and then, the hypoglossal nerves were visualized as it bifurcated with the lateral and medial divisions. Hx was made using sharp microfine forceps, and the incision was closed in a single layer by the use of 4‐0 Nylon sutures (Ethicon^®^, Edinburgh, UK). Sham surgeries were processed in an identical manner, but the nerves were not touched.

### Morris water maze

Morris water maze task was performed as described previously 9. Rats were subjected to the initial training sessions before the Hx or sham surgery in experiment 1 or at 1 week after the surgery in experiment 2 as the first phase of the experiments. In experiment 1, rats (*n* = 7–9) received the Hx or sham surgery on the following day of the initial training period, and then, a probe test was completed with the first trial on the 8^th^ day of post‐operational recovery. In experiment 2, Hx and sham rats (*n* = 9) were subjected to a probe test on the following day of the initial training period. The escape platform was removed from the tank. Then, the rats were placed in the opposite side of where the platform used to be located, and allowed to swim in the tank for 60 sec. In experiment 2, reversal training was followed for 3 consecutive days after the probe test. On the next day of the probe test, the platform was repositioned to the opposite quadrant from its original location within the maze, and all other conditions were the same as in the initial training trials. After the reversal training, rats were subjected to a probe test to confirm the strength of newly acquired spatial memory.

### Cresyl violet staining

On the 10^th^ day after the surgery, rats that are naïve from the behavioural tests were anesthetized with sodium pentobarbital (65 mg/kg), and the brain tissues were prepared as described previously 10. The hippocampal sections (between bregma −2.8 mm and −4.8 mm; 11) were coronally cut at 20 μm thickness with 240‐μm intervals between each section with Cryocut Microtome (Leica Microsystems, Bensheim, Germany) and collected in 0.1 M phosphate buffer, and mounted on gelatin‐coated glass slides, air‐dried. The tissue slides were dehydrated in a graded ethanol series, treated with chloroform‐ethanol solution (1:1) for 20 min., and then rehydrated. The brain tissues were briefly stained with 0.125% cresyl violet, dehydrated again and immersed in xylene for 20 min., and then cover slipped. Number of cells over the area of 3000 μm^2^ (100 μm length × 30 μm width) in CA1, CA3 and dentate gyrus (DG) were blind‐counted manually under a light microscope (Nikon, Tokyo, Japan). Eight hippocampal sections in each rat were counted (total 32–40 sections in each group, *n* = 4–5), and only cells with a clear cell body and prominent nucleus were counted as intact neurons.

### Western blot analysis

Rats that are naïve from the behavioural tests were killed by decapitation on the 10^th^ day after the surgery (*n* = 7–8), and the hippocampal tissues were rapidly dissected on ice, frozen in liquid nitrogen and stored at −80°C until used. Tissue samples were homogenized in cOmpleteTM, Mini Protease Inhibitor Cocktail (Roche Applied Science, Indianapolis, IN, USA) added T‐PERTM Tissue Protein Extraction Reagent (Thermo Scientific, Hudson, NH, USA) using bead homogenizer (Next Advance, Averill Park, NY, USA). The lysates were then centrifuged at 15682 g for 20 min. at 4°C, and the supernatant was collected and measured for protein content by Bradford assay (Bio‐Rad, #5000205, Hercules, CA, USA). Forty microgram protein samples were electrophoresed on 12% SDS‐PAGE gel and then transferred onto nitrocellulose membranes (Hybond‐C, Amersham, Bucks, UK). After blocking with 2% skim milk for 1 hr at room temperature, the membranes were incubated with primary antibodies for overnight at 4°C and then with the secondary antibodies for 1 hr at room temperature. Target proteins were detected using primary antibodies for BDNF (1:500, Millipore, Billerica, MA, USA #2064281), pMAPK (1:500, Cell Signaling, #9101s), and DCX (1:500, Abcam, Cambridge, UK, ab18723). Protein bands were detected using chemiluminescence substrate (Thermo Scientific, 34080) and imaged with a LAS‐1000 imaging system (Fuji film, Tokyo, Japan). Quantification of protein expression was conducted using ImageJ (NIH). Digitized values of each sample were normalized to the loading control β‐actin (1:2000, Sigma‐Aldrich, St Louis, MO, USA).

### Bromodeoxyuridine (BrdU) labelling

Rats received an intraperitoneal injection of BrdU (Sigma–Aldrich) at a dose of 150 mg/kg in 0.9% NaCl on the 10^th^ day after the surgery. BrdU injection was given daily for 3 consecutive days, and the rats were killed for immunofluorescence on the following day of the last injection (14 days after the surgery).

Hippocampal tissue sections were prepared at 40 μm thickness with Cryocut Microtome (Leica Microsystems) and the every third sections were collected in 0.1 M phosphate buffer, as described above. After washing 3 times in phosphate buffer for 5 min. each, tissue sections were denatured in 2 N HCl at 37°C for 40 min. and then rinsed twice in 0.1 M borate buffer (pH 8.5) for 5 min. After 1 hr of incubation in blocking solution [phosphate‐buffered saline (PBS) containing 2% normal horse serum and 0.3% TX‐100], sections were treated with mouse anti‐BrdU antibody (1:200; #MS‐1058, Thermo fisher scientific, Waltham, MA, USA) and rabbit anti‐NeuN antibody (1:500; ab104225, Abcam) for overnight at 4°C. After washing 3 times in PBS for 5 min. each, the sections were incubated with DyLight 488‐anti‐mouse IgG and DyLight 594‐anti‐rabbit IgG (1:1000; Vector laboratories, Burlingame, CA, USA) for 2 hr at room temperature. After washing three times in PBS for 5 min. each, coverslips were mounted using mounting medium with DAPI (Vector laboratories). Images of the slides were captured by confocal microscope (LSM‐700; Carl Zeiss Microscopy GmbH, Oberkochen, Germany), and BrdU‐labelled cells in dentate gyrus (20–24 brain sections/rat, *n* = 3) were blind‐counted manually.

### Doublecortin (DCX) immunostaining

On the 14^th^ day after the surgery, the hippocampal tissue sections were prepared as described above. Tissue sections at 40 μm thickness were collected in 0.1 M phosphate buffer with 240‐μm intervals between each section and incubated in blocking solution [phosphate‐buffered saline (PBS) containing 2% normal horse serum and 0.3% TX‐100] for 1 hr, and then with rabbit anti‐DCX antibody (1:500; ab18723, Abcam) for overnight. After washing 3 times in PBS for 5 min. each, the sections were incubated in DyLight 488‐anti‐rabbit IgG (1:1000; Vector laboratories) for 2 hr at room temperature. Coverslips were mounted using mounting medium with DAPI (Vector laboratories). Images of the slides (eight brain sections/rat, *n* = 3) were captured by confocal microscope (LSM‐700; Carl Zeiss Microscopy GmbH), and DCX‐positive cells in dentate gyrus were blind‐counted manually.

### Electrophysiology

On the 10^th^ day after the surgery, coronal slices (400 μm) from the transverse slices (400 μm) from the hippocampus were prepared as described previously 12. Slices were transferred to a holding chamber in an incubator containing oxygenated (95% O_2_ and 5% CO_2_) artificial cerebrospinal fluid for at least 1 hr before recording, and then were transferred to a recording chamber at 28–30°C, which was perfused with ACSF saturated with 95% O_2_ and 5% CO_2_ at a flow rate of 2 ml/ min. CA1 field excitatory postsynaptic potential (fEPSP) was evoked by SC stimulation (0.2‐ms current pulses) using a concentric bipolar electrode (200 μm diameter; FHC, Bowdoinham, ME, USA). Synaptic responses were recorded every 15 sec. with microelectrodes filled with ACSF (1–3 MOhm) and quantified as the initial slope of the extracellularly recorded excitatory postsynaptic potential (fEPSP) in CA1. Recordings were performed using an AM‐1800 microelectrode amplifier (A‐M systems, Sequim, WA, USA), a PG 4000A stimulator (Cygnus Technology, Delaware Water Gap, PA, USA), and a SIU‐90 isolated current source (Cygnus Technology). LTP was induced by theta‐burst stimulation, which consisted four trains of ten bursts (each with four pulses at 100 Hz). Only data from slices with stable recordings (<5% change over the baseline period) were included in the analysis. All data are presented as average ± S.E.M. normalized to the preconditioning baseline. The responses were digitized and analysed with IGOR software (Wavemetrics, Lake Oswego, OR, USA).

### Statistical analysis

Data were analysed by one‐ and two‐way analysis of variance (anova) and preplanned comparisons with the groups performed by *post hoc* Fisher's protected least significant difference (PLSD) test, performed with StatView software (Abacus, Berkeley, CA, USA). Synaptic plasticity at the hippocampal Schaffer collateral‐CA1 synapses was analysed by repeated‐measures anova. Significance was set at *P *<* *0.05, and all values were presented as means ± S.E.M.

## Results

### Morris water maze learning

**Figure 1:**
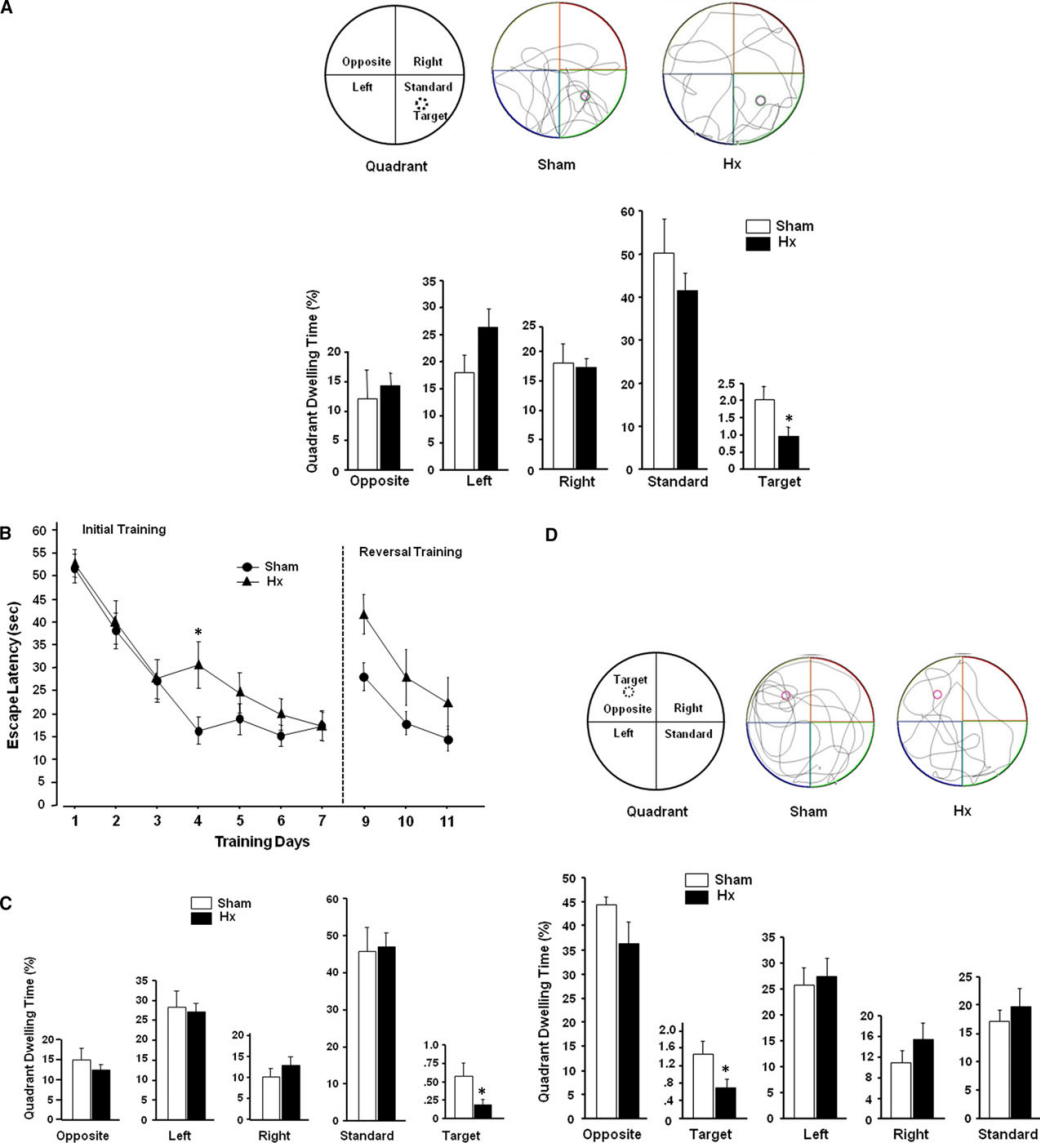
Morris water maze test. (**A**) Representative swim traces and goal quadrant dwell time during the probe test. Rats received Hx or sham surgery on the following day of the initial training, and then, the probe test was performed at a week after the surgery (**A**). Escape latencies during initial and reversal training (**B**), and goal quadrant dwell time during the probe test followed after the initial training (**C**) and the reversal training (**D**). Hx and sham rats were subjected to the initial training after a week of post‐surgical period (**B**), and then to a probe test on day 8 (**C**). Reversal training with repositioned platform was performed for 3 consecutive days from day 9 (**B**), which was followed by a probe test on day 12 (**D**). *P <* *0.05 *versus* sham, *n* = 7–9 in each group (**A**), *n* = 9 in each group (**B**–**D**), Values are presented as means ± S.E.M.

In experiment 1, two groups of rats assigned to Hx or sham surgery were subjected to the initial training of water maze task before the surgery. All rats showed a progressive decline in the escape latency over the 9 days of initial training. Mean escape latencies were 42.6 ± 2.794 sec. on day 1 and 16.9 ± 1.402 sec. on day 9 in rats assigned to sham surgery, and 46.7 ± 2.599 sec. on day 1 and 17.3 ± 1.415 sec. on day 9 in rats assigned to Hx surgery. Rats received Hx or sham surgery on the following day of the initial training period, and then, probe test was performed a week after the surgery to evaluate the Hx effect on memory retention. Hx or sham rats were allowed to swim for 60 sec. in the maze without the escape platform, and the time spent in each quadrant was recorded (Fig. 1A). Dwelling time in the target area, where the escape platform used to be placed, was significantly reduced in Hx rats (*P *=* *0.036) compared to sham rats.

In experiment 2, Hx and sham rats were subjected to the initial training sessions a week after the surgery (Fig. 1B). The mean escape latencies on the first training day did not differ between the groups (51.6 ± 3.1 in the sham group, 52.8 ± 3.0 in the Hx group); however, on the fourth day, a significant difference was found between the sham (16.3 ± 3.0) and the Nx (30.7 ± 5.1) group (*P *=* *0.031). Probe test was performed on the 8 days without the escape platform (Fig. 1C). Hx rats spent less time in target area, where the escape platform used to be placed, compared to sham rats (*P *=* *0.033). Rats were subjected to the reversal training for 3 consecutive days from day 9, in order to examine if the rats recall previously learned behaviour. The escape latencies of Hx rats to the platform in a new location were significantly longer than sham rats on day 9 (*P *=* *0.020), and analysis of two‐way anova (training days X surgery) revealed a main effect of Hx surgery [*F*(1,52) = 7.152, *P *=* *0.010] on the reversal training (Fig. 1B). In the probe test performed without the escape platform on the following day, dwelling time in the new target area was reduced in Hx rats (*P *=* *0.045) compared to sham rats (Fig. 1D).

### Cell numbers and BDNF expression in the hippocampus

**Figure 2:**
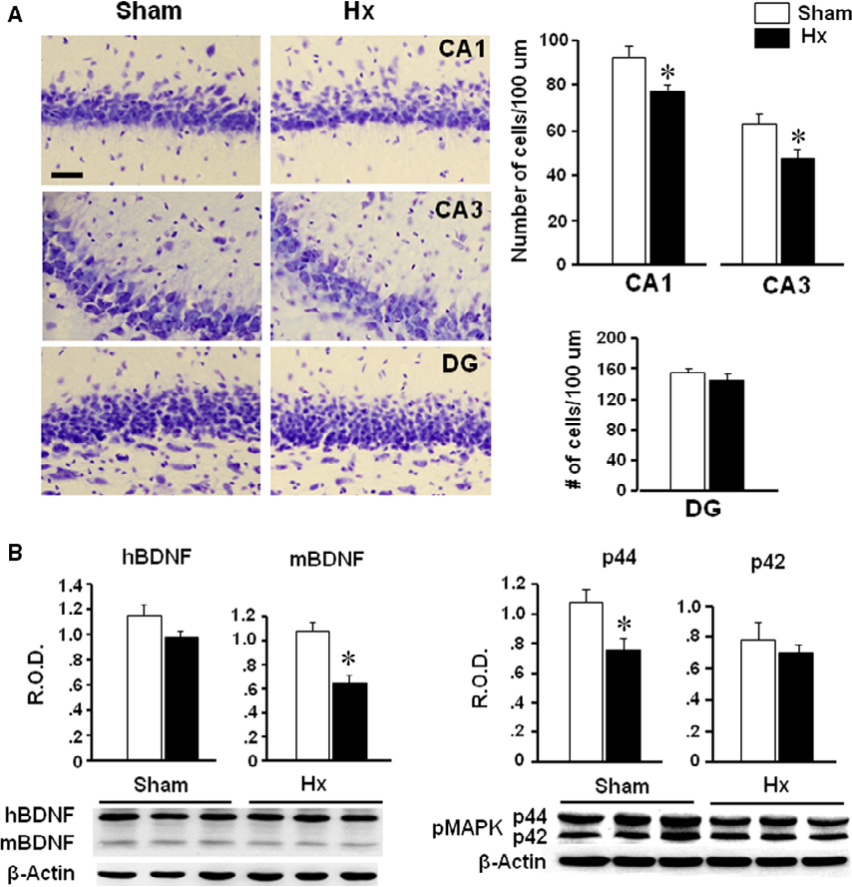
Representative photographs of cresyl violet staining and the number of cells stained in the CA1, CA3 and dentate gyrus (**A**), and BDNF and pMAPK Western blot analysis (**B**) of the hippocampus of Hx and sham rats. On the 10^th^ day after the surgery, Hx and sham rats that are naïve from the behavioural tests were killed for the cresyl violet staining (*n* = 4–5 in each group) or Western blot analysis (*n* = 7–8 in each group). *P <* *0.05 *versus* sham, DG; dentate gyrus, Scale bar; 20 μm. Values are presented as means ± S.E.M.

Hx and sham rats were killed on the 10^th^ day after the surgery to examine the hippocampal cell numbers and gene expression. The number of cells in the CA1 and CA3 regions, but not in dentate gyrus, of Hx rats was decreased significantly (*P *=* *0.0210 in CA1, *P *=* *0.0383 in CA3) compared to sham rats (Fig. 2A). Mature form of BDNF appeared to be reduced in the hippocampus of Hx rats (*P *=* *0.008) compared to sham rats (Fig. 2B). Also, pMAPK expression (p44, but not p42) was decreased in the hippocampus of Hx rats (*P *=* *0.032 *versus* sham).

### BrdU and DCX staining in the dentate gyrus

**Figure 3:**
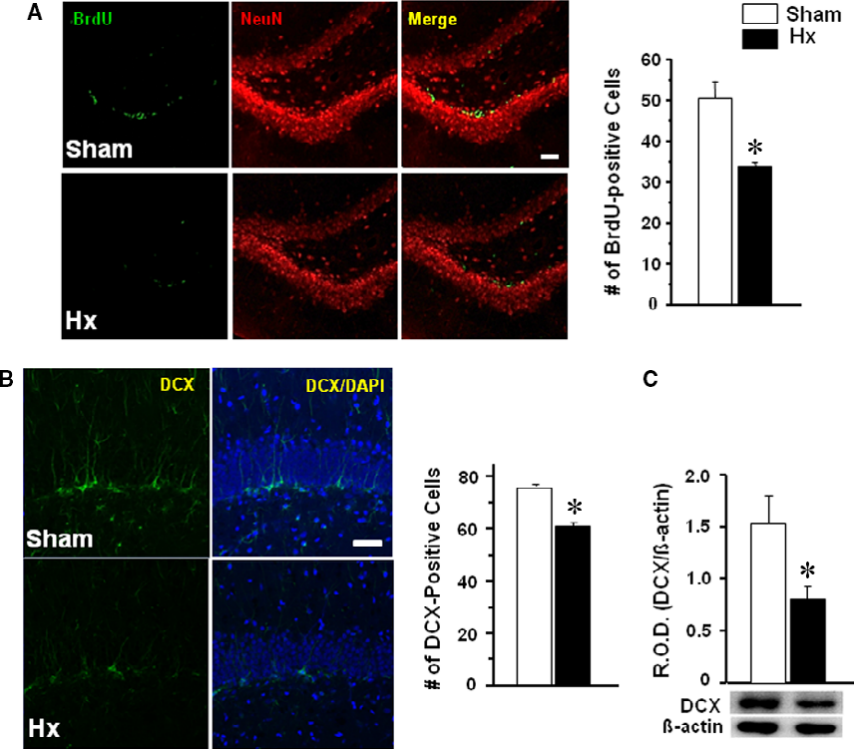
Representative photographs and quantification of BrdU (**A**) and DCX staining in the dentate gyrus (**B**), and Western blot analyses of DCX in the hippocampus (**C**) of Hx or sham rats. For BrdU labelling, rats received an intraperitoneal injection of BrdU daily for 3 consecutive days from the 10^th^ day after the surgery and then were killed on the next day of the last injection (*n* = 3 in each group, 20–24 brain sections/rat). Rats were killed on the 14^th^ day after the surgery for DCX immunostaining (*n* = 3 in each group, eight brain sections/rat) or Western blot analysis (*n* = 5–6 in each group). *P <* *0.05 *versus* sham, Scale bars; 50 μm. Values are presented as means ± S.E.M.

Rats were killed for immunofluorescence after a three consecutive daily injection with BrdU from the 10^th^ day after the surgery. The number of BrdU‐immunostained cells in the dentate gyrus was counted after double staining with NeuN antibody (Fig. 3A). BrdU‐positive cells were reduced in Hx rats compared with sham rats (*P *<* *0.0191). DCX immunostaining or Western blot analysis was performed after 14 days of post‐surgery (Fig. 3B). DCX‐immunostained cells in the dentate gyrus were significantly reduced in Hx rats (*P *=* *0.0012 *versus* sham) (Fig. 3B). Western blot analysis also showed a significant reduction in DCX in the hippocampus of Hx rats (*P *=* *0.0312) relative to sham rats (Fig. 3C).

### Hippocampal long‐term potentiation

**Figure 4:**
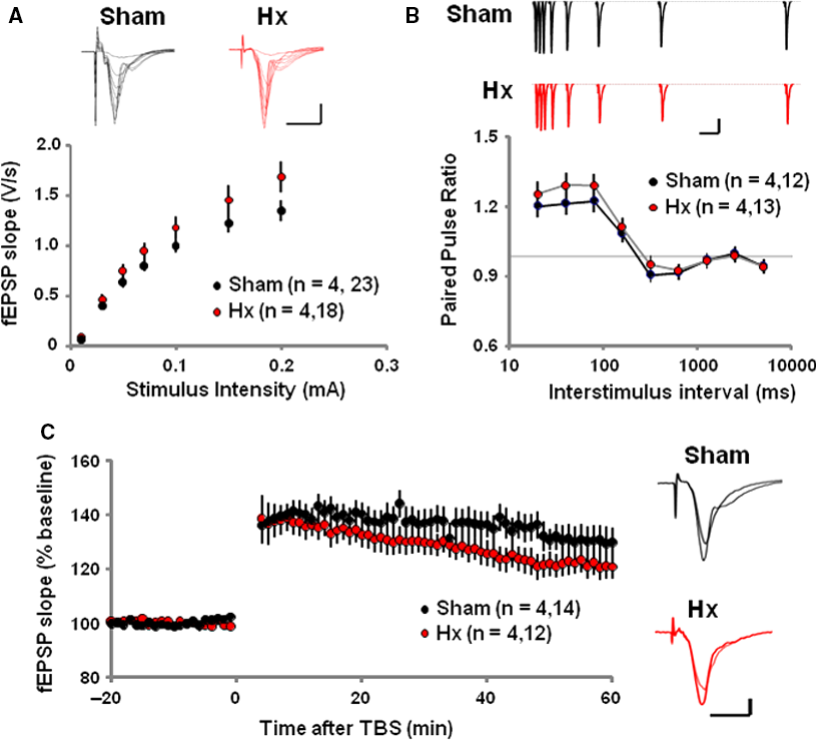
Impaired synaptic plasticity at the hippocampal Schaffer collateral‐CA1 synapses of Hx rats. (**A**) Average traces of fEPSPs (top) and summary graphs of Sham (Black) and Hx (red) rats. Scale bar; 10 ms and 0.5 mV. (**B**) Average traces (top) and summary graphs (bottom). Scale bar; 100 ms and 0.5 mV. (**C**) Average traces (Right) and summary graphs (Left). Long‐term potentiation (LTP) is impaired at hippocampal SC‐CA1 synapses in Hx rats. The magnitude of LTP is calculated by comparing the average slops of fEPSPs recorded during the last 10 min. with those recorded before stimulation. (Sham; 130.34 ± 5.1, Nx; 123.54 ± 2.8) (*P *<* *0.01, one‐way repeated‐measures anova). Scale bar; 10 ms and 0.5 mV, Individual points represent means ± S.E.M.

The effect of Hx on synaptic transmission and plasticity of hippocampus Schaffer collateral/commissural‐CA1 synapse was tested on the 10^th^ day after the surgery. The input–output relationship of AMPA‐R‐mediated field excitatory postsynaptic potential (fEPSP) responses was normal in Hx rats compared to sham rats (Fig 4A). Also Hx rats showed an intact paired‐pulse facilitation which is a form of presynaptic short‐term plasticity and implies normal presynaptic neurotransmitter release probability (Fig 4B). Interestingly, following the theta‐burst stimulation (TBS), fEPSP slopes recorded from the Hx hippocampal slices were significantly impaired compared to those recorded from the sham rats, implying impaired LTP induction in Hx rats (Fig 4C).

## Discussion

In the present study, Hx rats showed impairments not only in the retrieval of previously learned memory but also in the whole new learning and memory tasks during the water maze test. In the boundary of our knowledge, this is the first report demonstrating that tongue motor loss may affect hippocampus‐dependent cognitive function. Indeed, Hx‐induced impairments in spatial learning and memory were accompanied with reduced expression of BDNF in the hippocampus. The hippocampal BDNF is known to be implicated in learning and memory; that is, BDNF is essential to promote persistence of long‐term memory storage 13. Dysregulation of BDNF in the hippocampus has been proposed to underlie memory and cognitive disorders in human 14, 15. Genetic manipulation studies have shown that decreased levels of BDNF impair hippocampus‐dependent behavioural tasks such as spatial learning in the Morris water maze in rats and mice 16, 17, 18. Together, it is suggested that reduced BDNF expression in the hippocampus may play a role in the impaired cognitive function in Hx rats.

Brain‐derived neurotrophic factor is a neurotrophin in the nerve growth factor family expressed throughout the brain, including the hippocampus 19. BDNF affects all levels of cell generation, such as proliferation, survival, and differentiation in the hippocampus 20. In the present study, decreased BDNF expression in the Hx hippocampus was accompanied by decreased cell numbers in the CA1 and CA3 regions. Within the dorsal hippocampus, the CA1 and CA3 regions are important for memory retention. For example, atrophy of apical dendrites or reduced cell density in the CA1 and CA3 neurons leads to memory impairments in rats and mice 21, 22 and smaller hippocampal CA3/dentate volumes in humans are associated with learning and memory problems 23. Thus, it is likely that reduced cell numbers in the hippocampal regions, possibly due to decreased BDNF expression, are related with the cognitive dysfunction in Hx rats.

It was reported that exogenic BDNF increases BrdU‐labelled cells in the dentate gyrus of rats 24, suggesting its efficacy to stimulate neurogenesis. In the present study, the number of BrdU‐labelled cells was significantly decreased in the dentate gyrus of Hx rats, revealing decreased neurogenesis in the hippocampus. Neurogenesis occurs in the dentate gyrus of hippocampus, and the newly generated cells mature into functional neurons in the adult brain 25. In the present study, the level of doublecortin, a microtubule‐associated protein marker of migrating or immature neurons, was reduced in the dentate gyrus of Hx rats. Neurogenesis in the hippocampus has been suggested to be critical for certain types of learning and memory 26, and granule neurons in the dentate gyrus are produced throughout adulthood in animals and play a significant role in retention of hippocampus‐dependent learning and memory 27. Taken together, it is suggested that reduced neurogenesis in the dentate gyrus possibly due to reduced BDNF expression might have contributed to reduced cell numbers in the hippocampal CA1 and CA3 regions in Hx rats, which is a part of the neural mechanisms responsible for Hx‐induced cognitive impairments.

Impairments not only of adult neurogenesis but also of hippocampal synaptic plasticity associated with impairment of LTP are cellular mechanisms responsible for cognitive deficits 28. In the present study, the hippocampal LTP was impaired in Hx rats showing cognitive impairments with decreased BDNF expression in the hippocampus. BDNF is involved in LTP, a cellular model of learning and memory 29, and endogenic BDNF regulates excitability and synaptic plasticity of the hippocampal pyramidal neurons suggesting that BDNF modulates firing of hippocampal neurons 30. Thus, it is likely that decreased BDNF expression in the hippocampus of Hx rats may be responsible for the impaired LTP in relation with cognitive impairments.

BDNF is a canonical member of the neurotrophin family that has well‐described signalling pathways and mechanisms 31, 32, 33. Mature BDNF (mBDNF) preferentially binds to its receptor tyrosine kinase B, and initiates receptor dimerization and reciprocal phosphorylation of each receptor at tyrosine residues, which can activate three intracellular signalling cascades, such as the PI3K/Akt, phospholipase C and MAPK (microtubule‐associated protein kinase) cascades. Especially, mBDNF‐induced activation of MAPK cascade is believed to be involved in neuronal proliferation and differentiation. On the other hand, immature (homodimer) BDNF has a high affinity to p75 neurotrophin receptor, which triggers pro‐apoptotic signalling 34. In the present study, mBDNF and pMAPK (p44), but not hBDNF, were decreased in the hippocampus of Hx rats. Thus, it is suggested that neuronal proliferation and differentiation, rather than survival, in the hippocampus are affected by Hx. We have observed that the hippocampal expression of caspase‐3 did not differ between Hx and sham rats (data not shown), further supporting that Hx may not affect neuronal survival in the hippocampus.

Decreased mastication due to molar loss reduced BDNF expression in the hippocampus with impairment in hippocampus‐dependent learning task 35. Kondo and colleagues 35 suggested that decreased BDNF expression in the hippocampus of molarless mice may be a stress effect; that is, due to increased plasma glucocorticoids. However, the basal and stress‐induced levels of plasma glucocorticoids in Hx rats did not differ from sham‐operated control rats (our unpublished observation), suggesting that decreased BDNF expression in the hippocampus of Hx rats may not be a stress effect. As tongue movements are essential for an effective mastication, it can be still expected that impaired masticatory activity might have contributed at least partly to the impaired hippocampal cognitive function in Hx rats. A possible interaction between masticatory function and cognitive function in the hippocampus *via* modulation of dopamine responses has been suggested; that is, decreased mastication reduced the response of hippocampal dopamine neurons, impairing hippocampal learning ability 36, 37, and active mastication facilitated dopamine release in the hippocampus 38. Decreased BDNF signalling in the hippocampus was associated with reductions in dopaminergic and serotonergic neurotransmission in an animal model of behavioural impairments 39. A possible implication of dopaminergic and/or serotonergic neurotransmission in decreased BDNF expression in the hippocampus of Hx rats is currently under our investigation.

It should be noticed that decreased learning ability in molarless mice was not observed earlier than 2 months in young animals 37, 40; however, in the present study, decreased learning ability in Hx rats was observed in earlier time‐points; that is, a week after the surgery at the earliest time‐point. Thus, it is likely that the denervation effect, rather than the impaired masticatory activity as a secondary effect, is mainly in charge of the decreased learning ability in Hx rats. Sensory information from the oral cavity is transmitted through the trigeminal sensory nerve to the trigeminal sensory nuclei, cerebellum, hypoglossal motor nuclei and the brainstem reticular formation 41, 42, 43, 44, and may affect hippocampus *via* thalamus and cerebral cortex. We have observed atrophy in the tongue sensory epithelium; for example, reduced size and number of taste papillae, in Hx rats (our unpublished observation). The lingual, chorda tympani and glossopharyngeal nerves, which relay the somato‐/taste sensory information from the tongue, innervate to the brainstem nucleus tractus of solitarius (NTS) 1, and NTS is anatomically linked to the hippocampus *via* multisynaptic relays 45. Taken together, it is suggested that impaired relay of the oral sensory and motor information to brain may affect the hippocampal function and result in cognitive disorders in Hx rats. Further studies are warranted.

In conclusion, tongue motor loss impaired hippocampus‐dependent cognitive function. Decreased BDNF expression in the hippocampus appears to be implicated in its underlying molecular mechanism in relation with decreased neurogenesis, proliferation and impaired LTP in the hippocampus. As the Morris water maze used in this study was reported to be a sensitive assay for detecting Alzheimer's disease‐relevant impairments across species 46, the present study further emphasizes the importance of functional reconstruction after tissue removal in head and neck cancer patients.

## Conflict of interest

The authors confirm that there are no conflict of interests.
